# Molecules Affecting Brain Development and Nervous System

**DOI:** 10.3390/ijms24108691

**Published:** 2023-05-12

**Authors:** Kazuhiko Nakadate, Kiyoharu Kawakami

**Affiliations:** Department of Basic Science, Educational and Research Center for Pharmacy, Meiji Pharmaceutical University, 2-522-1, Noshio, Kiyose, Tokyo 204-8588, Japan; d236953@std.my-pharm.ac.jp

Brain development is the biological process through which neurons are produced. The processes contributing to neuronal development include proliferation, differentiation, migration, axon guidance, synapse formation, and neuronal network formation. Early postnatal brain development is important for general health in the long term. One primary reason for this is that the brain grows quickly before birth and continues to develop early postnatally. In humans, the first 8 years can lay the foundation for lifelong learning, health, and success. The brain continues to develop into adulthood. Neurons in the prenatal and postnatal brains respond to a variety of chemical signals. In the nervous system, chemical signaling has been demonstrated to be crucially involved in the development, normal functioning, and disorders of neurons and glial cells. In addition to genes, brain development relies on numerous factors, such as nutrition, toxins, and infections.

The human brain is significantly more complex than that of other animals, much of which develops rapidly after birth. Infants’ brain mass nearly doubles in the first 6 months, reaching 80% of its adult size by 2 years of age [1]. Other mammals are born with brains that approach the size and complexity of their adult brains. Even the closest evolutionary relative of humans, the chimpanzee, is born with a brain that is more than 40% the size of an adult brain [2], whereas the newborn human brain accounts for less than 30% of the size of an adult brain. It is speculated that a woman would need to be pregnant for 18–21 months for the brain of a human baby to develop similarly to that of a chimpanzee baby [3]. However, an infant’s head would grow too large to pass through the birth canal, which is the most likely reason why human babies are born with relatively underdeveloped brains.

Additionally, human babies are much more helpless and physically underdeveloped than most other mammals. They cannot stand and walk like calves and lambs, and they cannot live independently for a few months like puppies and kittens. In fact, babies continue to require love, care, and protection from their mothers and family members for many years. Since the human brain comprises 60% fat [4], fatty acids in breast milk and other nutrients are responsible for babies’ brain development. Fatty acids also have other benefits for the brain; the white matter connects brain areas and helps send signals throughout. Babies fed a diet rich in fatty acids, including breast milk, have been shown to have 20–30% more white matter than those fed infant formula. This extra white matter is concentrated in areas that control language, reasoning, emotion, and social and driving skills [5].

However, brain development begins in the fetal period; science has focused on the development of the brain and behavior, though the brain undergoes most of its basic morphological development in the womb. As such, it is appropriate to look more closely at fetal brain development concerning its impact on human development. There are many cases of autism spectrum disorder where signs of onset can be seen in infancy and adolescence. However, this predisposition is based on a broad developmental disorder of brain function, such as schizophrenia, in which the pathophysiological changes are thought to begin during the fetal period—an important time for understanding the etiology of neuropsychiatric disorders. Brain development is largely due to genetic programming. However, the developing brain is dynamically influenced by the environment, and the onset of functional brain developmental disorders, such as autism and schizophrenia, is based on genetic predisposition and environmental interactions [6]. Environmental factors are thought to modify gene expression, amplify abnormalities, and affect pathogenesis.

Due to the high concordance rate (80–90%) in identical twins with functional brain development disorders, such as autism and schizophrenia, the etiology is thought to be mostly related to genetics. Surprisingly, a large-scale epidemiological and mathematical modeling study of monozygotic and dizygotic twins [7] recently reported that environmental factors (58%) had a stronger effect than genetic factors (38%) on the development of autism. Moreover, because the incidence rate in dizygotic cases was higher than that in fraternal cases, the influence of the intrauterine environment was thought to be stronger than that of the postnatal environment. The concordance rate of schizophrenia in monozygotic twins was found to be approximately 50%, which was lower than for autism, and it has long been known that the influence of environmental factors is predominant in schizophrenia [8]. Issues in the intrauterine environment seem to have significant effects on these disorders. A large body of evidence has demonstrated a relationship between various stressors during the fetal period (e.g., maternal mental stress, infections, and malnutrition) and these diseases [9]. Furthermore, there is also abundant epidemiological and pathological evidence for abnormalities in inhibitory neurons and the neurotransmitter gamma-aminobutyric acid (GABA) in the brains of those with these disorders [10,11]. However, the mechanism by which maternal stress, infection, and nutritional status affect the fetal GABA system remains to be elucidated. If a causal relationship could be established between various environmental factors, such as intrauterine stress, and abnormalities in fetal GABA systems, it would be a considerable contribution to research on babies, including fetuses, from a biological perspective.

The development of this neural circuitry begins in the womb, continuing after birth. Activity-dependent changes (plasticity) in neurons are considered the basic mechanism of neural circuit formation and learning. Neural circuit formation in the developing brain occurs through interactions between genetic and environmental factors. During a critical period, neural circuits are reconfigured through an experience-dependent manner, and brain functions are subsequently determined. Neurons in the cerebral cortex are broadly classified into two types: excitatory and inhibitory neurons. Inhibitory neurons that use GABA as a neurotransmitter account for approximately 20% of all neurons. Excitatory cells release glutamate as a transmitter to depolarize postsynaptic cells, whereas inhibitory cells release GABA to hyperpolarize postsynaptic cells and regulate information transmission in excitatory neurons. It has been suggested that GABA cells play an important role in the expression of plasticity in the developing visual cortex [12], and the relationship between GABA cells and the expression of plasticity has received much attention. Although little is known about how hyperpolarization induces plasticity [13], in certain parts of the brain, it induces oscillations (oscillation and rhythmic activity) in inhibitory neuronal circuits, accompanying the activity of excitatory synapses. For example, it has been suggested that wave breaking and spindle waves recorded by electroencephalography are generated via the oscillation of the thalamocortical circuit at the onset of non-rapid eye movement during sleep and are thought to be connected with memory formation and epileptic seizures.

In addition to the neurotransmitter GABA, various other neurotransmitters are involved in the formation, reorganization, and maintenance of neural circuits. Changes in the release of glutamate, a major neurotransmitter, and AMPA receptors, a glutamate receptor, are essential for the formation and maintenance of neural circuitry [14,15,16]. Furthermore, serotonin [17,18], noradrenaline [17,19], and acetylcholine [18,20] are involved in the regulation of neural networks and are vital for the establishment of memory and learning. It has also been suggested that amine system components, such as serotonin and GTP-cyclohydrolase 1 (an enzyme essential for amine biosynthesis) [21,22], are important in psychological development.

It is of the utmost importance to elucidate the mechanism of critical period expression, as it is the time for both neural circuit adjustment during postnatal development and the development of brain neural circuits during the fetal period. Furthermore, revealing regulatory molecules in this context will have a significant impact on broader society. It is conceivable that this will lead to a debate on how early and lifelong education should be implemented. If we can find a way to control the development of neural circuits and their expression during critical periods of postnatal development, the impacts will be even greater. If the expression of plasticity can be controlled, it will be possible to administer effective medications for treating related disorders. Furthermore, the development of dementia-improving drugs must be considered.
